# Influence of heat transfer and wetting angle on condensable fluid flow through nanoporous anodic alumina membranes†

**DOI:** 10.1039/d2cp04577j

**Published:** 2023-01-10

**Authors:** Thomas Loimer, Stepan K. Podgolin, Javad Sodagar-Abardeh, Dmitrii I. Petukhov, Andrei A. Eliseev

**Affiliations:** a Institute of Fluid Mechanics and Heat Transfer, TU Wien Vienna Austria thomas.loimer@tuwien.ac.at +43 1 58801 32233; b Department of Materials Science, Lomonosov Moscow State University Russia; c Department of Chemistry, Lomonosov Moscow State University Moscow Russia

## Abstract

The flow of isobutane and of freon 142b (1-chloro-1,1-difluoro-ethane) through anodic alumina membranes with pore diameters between 18 and 60 nm in a capillary condensation regime is experimentally and theoretically explored. The capillary condensation effect increases the membrane permeance for condensable gases from 25 to 150 m^3^(STP) m^−2^ bar^−1^ h^−1^ at certain conditions. To describe the experimental results, a model is suggested accounting for heat transfer from the condensing to the evaporating meniscus, different boundary conditions for the heat transfer between the environment and the membrane, and wettability of the pore wall. The proposed model indicates a large influence of heat supply from the environment to the membrane on the permeance in the capillary condensation regime and a moderate influence of condensate contact angle in the range of 0–60°. Measuring the temperature of the permeate side of the membrane allows to find a suitable boundary condition to describe heat transfer. The obtained boundary condition yields an excellent fit of experimental results of condensate flow through membranes with different pore diameters for the two utilized fluids. Also, confocal Raman spectroscopy gave evidence on the fraction of pores filled with condensate.

## Abstract 2

The flow of isobutane and of freon 142b (1-chloro-1,1-difluoro-ethane) through anodic alumina membranes with pore diameters between 18 and 60 nm in a capillary condensation regime is experimentally and theoretically explored.

## Graphical abstract

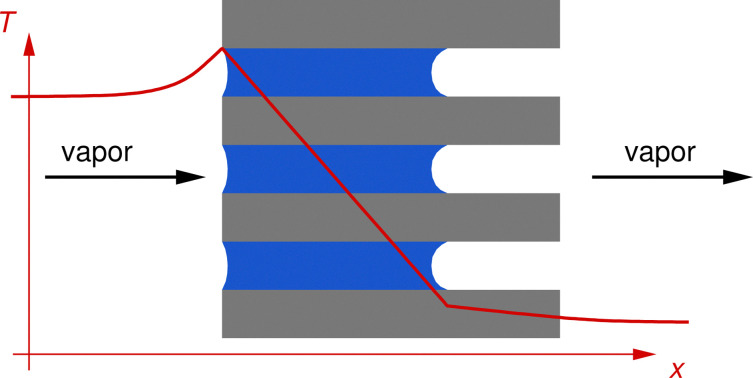

## 1 Introduction

The processes of vapor transport and liquid-vapor phase change in nanoporous media play an important role in various applications, *e.g.*, air gap water distillation,^1^ cooling of high power electronics,^2^ or separation of technological and natural gas mixtures.^3,4^ The flow of vapors in the capillary condensation regime has been studied by several authors since the pioneering works of Rhim and Hwang^5^ and Lee and Hwang.^6^ Rhim and Hwang used Kelvin's equation to estimate the capillary condensation pressure and the Young–Laplace equation for the calculation of the pressure difference in the condensed phase. These authors postulated that the enthalpy of vaporization released at the condensing meniscus is transported by heat conduction to the evaporating meniscus. Hence, regardless of the boundary conditions, a temperature variation must exist within the membrane.^5^ Later, several authors^7–10^ assumed an isothermal flow through the nanoporous media, irrespective of whether phase changes take place or not. In an extensive review,^11^ the description of the flow based on the Young–Laplace and Kelvin equations was termed “classical capillary theory”. Throughout this review, a temperature variation is not considered for the description of capillary condensation. Typically, utilization of “classical capillary theory” for vapor flow description does not take into account considerations about a temperature profile along the pore.^12–14^

Another approach^15^ is based on describing the flow of a vapor near saturation through nanoporous media assuming Darcy's law, allowing a temperature distribution and taking into account the energy balance as well as the real gas properties of the vapor, such as the realization of the Joule Thomson effect. However, capillary effects given by the Young–Laplace or Thomson–Kelvin equations are not considered. According to this approach,^15^ if the membrane permeability is below a critical value determined by the thermodynamic quantities, the energy balance requires condensation of the fluid in the membrane. In subsequent works, attempts were made to modify classical capillary condensation theory by taking into account heat transport between condensing and evaporating meniscus and the energy balance.^16–18^ It was found that allowing a temperature distribution and heat flux gave significantly different results under capillary condensation conditions than an isothermal description of the process.^17^ However, in our previous work^18^ we demonstrate that both adiabatic and diabatic boundary conditions do not allow to correctly fit the obtained experimental data on isobutane permeance through the anodic alumina membrane in capillary condensation regime. A properly chosen heat transfer coefficient gave a better fit to the experimental data. In the present work, we reveal the real boundary conditions based on the results of direct measurements of membrane permeate side temperature. To the best of our knowledge, the membrane temperature measurements in the capillary condensation regime have been performed only in one previously published work.^17^ However, the obtained temperature distribution did not prove the authors’ assumption on the adiabatic boundary condition. An overview of literature on permeability measurements in capillary condensation regime for different membranes and the models utilized for description is given in Table 1.

**Table 1:** Consolidated data on permeation measurements in capillary condensation regime

Membrane material	Substance	Pore size (nm)	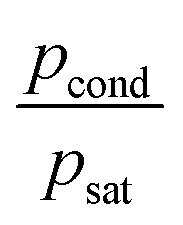 a	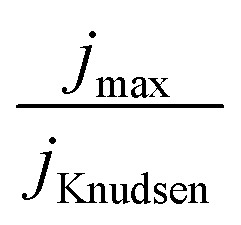 b	Thermodynamic model	Ref.
Vycor glass	*n*-Butane	8	0.773	4.3	Isothermal	19
	Water	8	0.769	1.5	Isothermal	6
	Freon-113	8	0.82	1.8		
	*n*-Butane	8	0.773	5.7	Isothermal	9
	Freon-112	8	0.83	≈2		
	Propane	8	0.89	≈9		
	*n*-Butane/	20	No data	4	Comparison of isothermal and adiabatic desc.	20
	Isobutane	30	No data	4		
Polypropylene	Water	370	No data	No data	Accounting for energy balance, thermal conductivity, vaporization enthalpy	21
Ceramic	Propylene	3	0.67	1.7	Isothermal	7
alumina						
Anodic	Isobutane	20	0.88	3.0	Isothermal	10
alumina		40	0.90	2.0		
membrane		70	0.97	1.7		
		90	0.96	1.9		
Track-etched	Nitrogen	10	0.9	≈50	Isothermal	8
polycarbonate	Oxygen	10	0.9	≈50		

a *p*_cond_ – minimum pressure at which condensation occurs; *p*_sat_ – saturation pressure.

b *j*_max_ – maximum measured mass flux; *j*_Knudsen_ – mass flux due to molecular flow.

Generally, vapor transport near saturation pressure through nanoporous media includes the following stages: condensation, transport in a liquid phase, evaporation, and transport in the gaseous phase. The transport through nanoporous media both for the liquid phase and for the gas phase can be comparatively well described by the Poiseuille^22^ and Knudsen^23^ equations, respectively. At the same time, the influence of condensation and evaporation rate is poorly described in the literature. Intuitively, based on the Laplace equation, the variation of pore wall wettability may have a significant impact on capillary pressure, which determines condensation/evaporation rate and the permeance of porous media in the capillary condensation regime. Therefore, several works paid attention to these important factors. Several calculation approaches, such as molecular dynamic^24,25^ or Monte Carlo simulation^26,27^ have been utilized for the description of evaporation from nanoporous media, taking into account surface–liquid interaction or real temperature and pressure conditions. However, these methods fail to accommodate the macroscopic properties of fluids. Lattice–Boltzmann methods seem to be a promising approach to bridge the gap between microscopic and macroscopic scales.^28,29^ At the same time, the experimental validation of theoretical results remains an open question because the early published experimental results on evaporation and condensation in nanochannels are controversial. In ref. 30 it is shown that capillary condensation of propane in a 8 nm channel could be described with Kelvin's equation and a continuum description. At the same time, other authors^31^ demonstrate that the wettability has a significant influence on the water evaporation flux – channels with hydrophilic surface demonstrate an evaporation rate 11 times higher than the fluxes predicted by the Hertz–Knudsen equation. In contrast, the evaporation rate from the hydrophobic nanopores attains only 66% of predicted values. The main part of the published works describe the process where only a single phase transition – condensation or evaporation – occurs in a nanochannel. We are able to find only one manuscript where the study of wettability on the vapor transport in a capillary condensation regime was reviewed.^12^ The classical theory of water leak rates at high humidities has been extended by variable curvature of condensing meniscus. This allows explaining a huge increase of vapor flux in the capillary condensation regime and estimating the contact angle value above 75° for the alumina wall by fitting experimental results.^12^ However, the authors consider capillary condensation as an isothermal process, which can significantly distort the obtained results.

Thus, in the present work, we suggest a model considering heat transfer from the condensing to the evaporating meniscus, different boundary conditions for the heat transfer from the environment to the membrane, and different wettability of the pore wall with condensate. A one-dimensional description of the flow process is given since the length of one pore, 0.1 mm, is large compared to the lateral distance to the next pore. The latter is of the same order as the pore diameter, 18 to 60 nm. Edge effects from the boundaries of the membrane samples are also not taken into account, since the diameter of the membranes, 16 mm, is much larger than the membrane thickness. The influence of contact angle and heat transfer on the calculated values of permeance in the capillary condensation regime is analyzed. An experimental study of anodic alumina permeance in a capillary condensation regime is performed using two vapors with quite different properties: isobutane and 1-chloro-1,1-difluoroethane (freon 142b). The boundary conditions for heat transfer have been found from the measurements of permeate side temperature of the membrane. The determined boundary conditions allow making an excellent fit of the obtained experimental data. Also, analysis of the fitted curves indicates a different wettability of the membrane pore wall with isobutane and with freon 142b.

## 2 Experimental

**Fig. 1:**
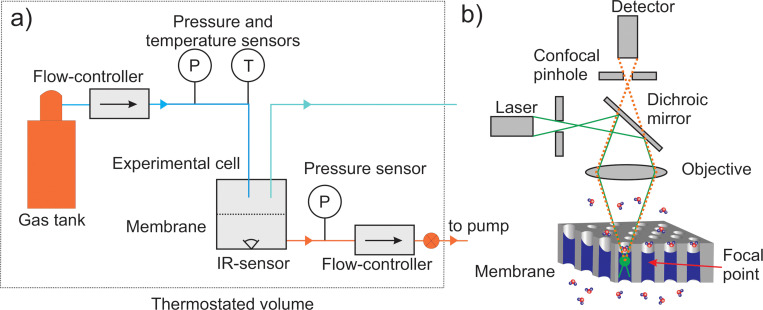
(a) Experimental setup to measure membrane permeance. (b) Scheme of confocal Raman spectroscopy study to determine the amount of liquid within the membrane.

Porous anodic alumina membranes with an average pore diameter from 18 nm to 60 nm were obtained using a procedure described earlier.^32^ The anodization of electropolished aluminum was carried out in a two-electrode cell in 0.3 M H_2_SO_4_ for 25 V voltage or in 0.3 M H_2_C_2_O_4_ for 40 V and 80 V. After anodization, the remaining aluminum was selectively dissolved in 0.5 M CuCl_2_ in 5 vol% HCl, followed by removing the barrier layer by chemical etching in 25 vol% H_3_PO_4_ aqueous solution with electrochemical detection of the opening of the pores.^22^ The average pore diameter and porosity of obtained membranes were characterized using scanning electron microscopy (Leo Supra 50VP) with subsequent image processing in ImageJ software. Isobutane and 1-chloro-1,1-difluoroethane (freon 142b) permeance for obtained membranes was measured by registering gaseous flux with mass flow controllers SLA5850 (Brooks, USA) and measuring pressure at the feed and the permeate side, simultaneously with measuring the saturation pressure on the gas vessel with Carel SPKT pressure transducers, see Fig. 1(a). This measurement technique was previously described in detail in ref. 10, 23 and 33. The typical experiment was started from 1 bar at the feed and the permeate side of the membrane. The feed pressure was increased step by step until the feed pressure reached the saturation pressure. The typical duration to establish steady flow conditions after a change of feed side pressures did not exceed 60 min. The experimental cell was placed into a thermostat (Huber) to minimize the temperature variations during measurements. The design of the experimental cell is depicted in Fig. S1 in the ESI.† For one experimental run, the temperature of the permeate side of the membrane during the permeance measurements was monitored by IR sensor MLX90614 (Melexis, China).

Confocal Raman spectroscopy performed on a Renishaw InVia spectrometer equipped with Leica DMLM optics (50× objective) and 20 mW 532 nm solid-state laser was utilized to investigate the degree of filling of the membrane with condensate in the capillary condensation regime. During the measurements the membrane was placed in a custom-designed cell with a quartz window, enabling acquisition of Raman scattering and allowing to vary feed and permeate side pressure in a wide range. The degree of filling was determined by scanning the intensity of C–F vibrational mode with a 2-micron depth increment, see Fig. 1(b). Experimental data were processed using Wire 3.4 Renishaw software.

## 3 Theory

The flow of vapours through anodic alumina membranes is described as the one-dimensional flow of a fluid trough a bundle of parallel, round pores. With exception of some downstream boundary conditions, the flow model is described in more detail in a previous work.^17^ Nevertheless, here below the main properties of the flow model are given.

**Fig. 2:**
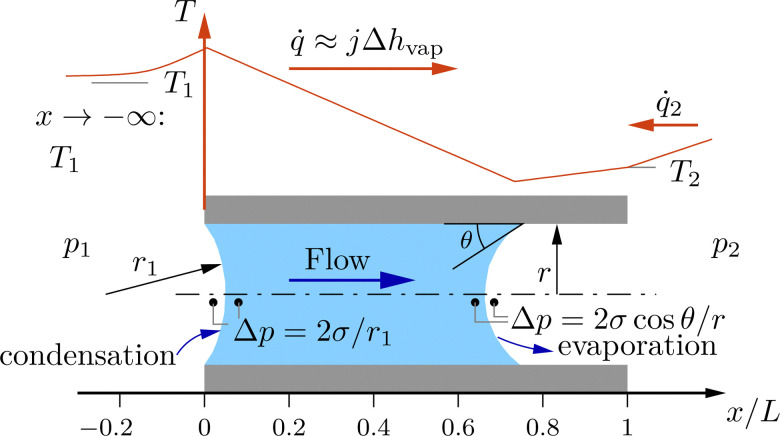
Schematic of the one-dimensional model. Liquid phase is indicated by blue color, vapor phase remains blank. On top, a possible temperature distribution and corresponding heat fluxes are indicated.

The flow resistance is caused by viscous forces for the liquid phase of the fluid, while for the gaseous phase of the fluid it is caused by a combination of viscous and molecular flow. At interfaces between the phases, the pressure difference across the curved menisci is given by the Young–Laplace equation, see Fig. 2. Conforming to the one-dimensional description, heat is conducted only in longitudinal direction. The temperatures of the fluid and the membrane material are the same and an effective thermal conductivity of the combined fluid and membrane material is used.

In case of condensation, the major part of the enthalpy of vaporization released at the condensing interface is transported by heat conduction to the evaporating interface. A small part of the enthalpy of vaporization may be transported by heat conduction in the upstream direction thereby forming an upstream thermal boundary layer. Since the upstream temperature boundary layer decays exponentially, the spatial domain formally extends to minus infinity. In contrast, heat conduction from the downstream side of the membrane would lead to an exponentially growing temperature distribution. Therefore, the domain ends at the downstream front of the membrane and a boundary condition on temperature, or heat flux, or on a combination of both is required at that position. Fig. 2 shows the sketch for a temperature distribution with some given heat flux supplied from the environment to the downstream side of the membrane.

The equations governing the flow are the balances of mass, momentum and energy,^17^

1 *j* = constant

2
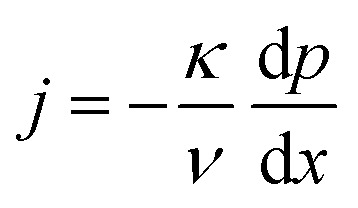

3 *jh* + *q̇* = constant

where *j* is the mass flux, *κ* is the permeability of the membrane, *ν* denotes the kinematic viscosity of the fluid, *p* refers to the pressure, the spatial coordinate in flow direction is given by *x*, the specific enthalpy of the fluid is given by *h* and *q̇* is the heat flux. The equations are valid for both the liquid and the gaseous phase of the fluid. The appropriate substance properties, *e.g.*, for the kinematic viscosity, must be used. The balance equations are supplemented by the thermal and caloric equations of state, by Fourier's law of heat conduction to express the heat flux and by the Young–Laplace and Kelvin's equations at interfaces between liquid and gaseous flow domains.^17^ At the meniscus in the inside of the membrane where the radius of curvature is given, *cf.*Fig. 2, Kelvin's equation is a condition for the location of the interface. For a plane interface simply the saturation pressure could be used, and the flow would be integrated in the liquid or gaseous state until the saturation pressure is reached. Kelvin's equation here accounts for the curvature. Differently, for the meniscus at the upstream front of the membrane the location is given, and the curvature of the meniscus is determined from Kelvin's equation.

The system of governing equations is completed by the boundary conditions. Far upstream, the pressure and temperature must be equal to the upstream pressure and upstream temperature, *p*_1_ and *T*_1_, respectively, where the upstream state is denoted by 1,

4 *x* → −∞: *p* = *p*_1_, *T* = *T*_1_, *q̇* = 0

At the downstream front of the membrane the pressure is given,

5 *x* = *L*: *p* = *p*_2_

where *L* is the thickness of the membrane and 2 refers to the state at *x* = *L*, which is the downstream state. For the temperature at the downstream front of the membrane, a number of boundary conditions are possible. One is the adiabatic boundary condition, demanding zero heat flux between the environment and the downstream side of the membrane,

6 *q̇*_2_ = 0

In this case, from the energy and the continuity equation the enthalpies far upstream and downstream stay the same, *h*_1_ = *h*_2_. The temperature difference for the adiabatic boundary condition is denoted by *T*_1_ − *T*_2,adiabatic_ and can be computed from integration of the Joule–Thomson coefficient,7
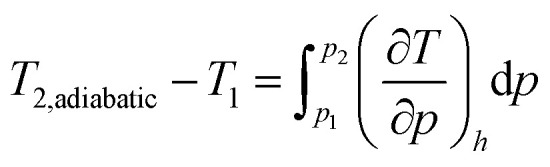
The diabatic downstream boundary conditions sets the downstream temperature equal to the upstream temperature,

8 *T*_2_ = *T*_1_

The rationale for the diabatic boundary condition is that the experimental cell is placed into an isothermal bath, and the mass and the heat capacity of the cell are much larger than the mass of fluid permeating through the cell during a considerable time span. Hence, the upstream temperature *T*_1_ is regarded as a very good estimate for the temperature of the environment. Raising the downstream temperature from *T*_2,adiabatic_ to *T*_1_ requires a certain heat flux from the environment to the downstream side of the membrane, which can be computed from the caloric equation of state of the fluid.

After performing a few experiments, a boundary condition that was found to best describe the condition measured at the downstream side sets the temperature difference to a fraction of the temperature difference expected in an adiabatic process,

9 *T*_1_ − *T*_2_ = *ϕ*(*T*_1_ − *T*_2,adiabatic_)

where *ϕ* is a number between zero and one. A value of one is equivalent to the adiabatic b.c., eqn (6), zero corresponds to the diabatic boundary condition, eqn (8).

If the heat transfer to the downstream side of the membrane can be described by a heat transfer coefficient, the boundary condition is

10 *q̇*_2_ = −*α*(*T*_1_ − *T*_2_)

where *α* denotes the heat transfer coefficient. Here, again, *T*_1_ is regarded as the temperature of the environment.

From Fourier's equation and the energy equation, the characteristic length scale for the temperature variation at the feed side is11
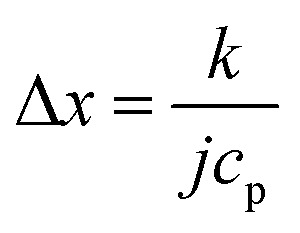
where *k* is the thermal conductivity of the fluid and *c*_p_ refers to the specific heat capacity. For gaseous flow through the entire membrane, the mass flow density can be estimated from eqn (2), and the characteristic length scale becomes12
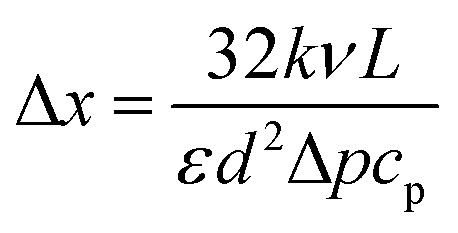
where *ε* is the void fraction of the membrane and *d* denotes the pore diameter. An estimate for the characteristic length scale yields Δ*x* = 3 mm, where approximate values of *k* = 0.01 W m^−1^ K^−1^, *ν* = 2 × 10^−6^ m^2^ s^−1^, *L* = 0.1 mm, *d* = 40 nm, *ε* = 0.15, Δ*p* = 10^5^ Pa and *c*_p_ = 10^3^ J kg^−1^ K^−1^ were used. This decay length is much larger than the membrane thickness, but smaller than the dimensions of the feed side volume of the permeation cell.

## 4 Computational

After substituting for the heat flux from Fourier's law in eqn (3), eqn (1)–(3) yield a system of two ordinary differential equations of first order in the variables *p* and *T*. Eqn (2) and (3) are integrated as an initial value problem, with the mass flow density *j* as a parameter.

It was found beneficial to integrate the governing equations against the flow direction in upstream direction, starting from the downstream side of the membrane at *x* = *L* where the downstream boundary conditions are applied. The governing equations are integrated varying the mass flow density *j* until the upstream pressure obtained by integration was equal to the required *p*_1_ within a tolerance of (*p*_1_ − *p*_2_)/1000. Integrating in downstream direction from the upstream state would require an additional iterative loop to determine the upstream temperature layer.

Computations were carried out with a matlab-program (MATLAB Release 2020a. The MathWorks Inc., USA). The program code is freely accessible,^34^ as well as all experimental data and the program files that served to produce the figures contained in this publication.^35^ Integration was done with a high-order Runge–Kutta scheme, the ode45() builtin function. All necessary material properties were taken from engineering correlations and implemented as temperature and possibly pressure-dependent functions in matlab. A virial equation with the first virial coefficient given by a temperature dependent function was used as the thermal equation of state. The integrability conditions from the second law of thermodynamics or Clausius–Clapeyron equation were used to determine thermophysical properties of a substance, if applicable.

At the upstream front, if the pressure is larger than the saturation pressure *p*_sat_, a liquid film forms in front of the membrane. Since there is a temperature variation in the liquid film, at a certain film thickness *p* = *p*_sat_ holds, this is the location of the surface of the liquid film. Upstream of the liquid film there is gaseous flow with an exponentially decaying temperature variation if *p*_1_ ≠ *p*_sat_(*T*_1_). Otherwise, if at the upstream front of the membrane the pressure is smaller than the saturation pressure, curved menisci form at the entries of the pores and Kelvin's equation yields the curvature of these menisci.

## 5 Results and analysis

### 5.1 Downstream boundary condition

**Fig. 3:**
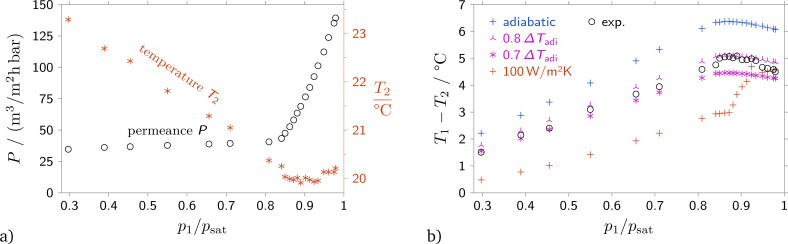
(a) Simultaneous measurement of permeance and downstream temperature. (b) Measured and expected temperature differences for three different boundary conditions: adiabatic, *T*_1_ − *T*_2_ = 0.7(*T*_1_ − *T*_2,adiabatic_) and *q̇*_2_ = −*α*(*T*_1_ − *T*_2_), where *α* = 100 W m^−2^ K^−1^. Pore size 40 nm, isobutane.

In order to compare the predictions from the suggested model to experimental data, the correct downstream boundary conditions must be used. While the upstream state and the downstream pressure were determined for all experiments, data for the downstream temperature or the downstream heat flux was not readily available. In order to resolve the problem, for one experiment with a 40 nm anodic alumina membrane the temperature of the downstream side of the membrane was measured with an IR sensor, see Section 2. The experiment was performed with simultaneous measurement of the flow rate, pressures and the downstream temperature, see Fig. 3(a).

**Fig. 4:**
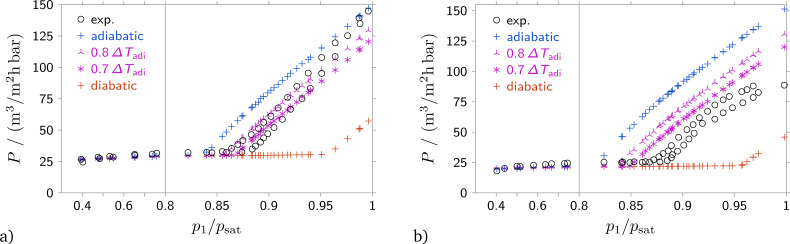
Permeance *versus* relative upstream pressure for a pore size of 40 nm, contact angle *θ* = 0°. Data is shown for a different membrane than that used in Fig. 3. Flow of (a) isobutane and (b) freon 142b.

Comparing the measured temperature difference with the temperature difference expected from the Joule–Thomson effect, it was found that the measured temperature difference is between 20% and 30% below the value predicted from the Joule–Thomson effect, see Fig. 3(b). However, since temperature data is not available for all experiments, a suitable boundary condition should be chosen. Therefore, conferring to permeance data, Fig. 4 and Fig. S2, S4 in the (ESI†), eqn (9) with a value of *ϕ* = 0.7 was used as boundary condition for all comparisons with experiment.

Regarding the heat flux from the downstream side necessary to sustain the required temperature, eqn (9) can be rewritten as *T*_2_ = *T*_2,adi._ + (1 − *ϕ*)(*T*_1_ − *T*_2,adi._). Remembering that *h*(*T*_2,adi._,*p*_2_) =*h*_1_, the downstream specific enthalpy can be estimated by *h*_2_ = *h*_1_+ (1 − *ϕ*)*c*_p_(*T*_1_ − *T*_2,adi._), assuming constant *c*_p_. Substituting into the energy equation, the boundary eqn (9) is equivalent to

13 *q̇*_2_ = −*j*(1 − *ϕ*)*c*_p_(*T*_1_ − *T*_2,adiabatic_)

Hence, with *T*_1_ − *T*_2,adiabatic_ being proportional to *p*_1_ − *p*_2_, we have the result that the heat flux supplied to the downstream front of the membrane is proportional to the mass flow density *j* times the applied pressure difference.

### 5.2 Description of measurements using suggested model

The suggested model was utilized to make predictions of the permeance using different boundary conditions for the heat transfer between the environment and the backside of the membrane. The predictions for the permeance were compared with experimental data, see Fig. 4. The influence of the wetting angle on the membrane permeance in the capillary condensation regime was not taken into account at this stage, and the value of the contact angle was set to zero. To compare the experimental data with computational results, the permeances were calculated for the same upstream pressure and upstream temperature and the same downstream pressure as was realized in the experiment. For each measurement, three computations with three different downstream boundary conditions were performed, adiabatic, eqn (6), diabatic, eqn (8), and boundary condition (9) with *ϕ* = 0.7. While Fig. 4 shows a comparison of experimental data and computations for a membrane with 40 nm, data for membranes with pore sizes of 18 nm and 60 nm are shown in Fig. S2 and S4 in the ESI.†

The permeance-pressure dependence was measured for the feed stream pressure increasing from 0.4*p*_sat_ to *p*_sat_ and then for decreasing pressure. The graphs of the permeance *versus* the relative feed stream pressure, *p*_1_/*p*_sat_, show several distinct features. For *p*_1_/*p*_sat_ smaller than about 0.9, the permeance stays nearly constant. In this region, the permeance slightly increases with increasing upstream pressure. The second peculiar feature is the strong and sudden increase of the permeance with increasing upstream pressure for *p*_1_/*p*_sat_ larger than about 0.9. The third feature is the hysteresis loop, which is quite similar to the hysteresis observed in type IV adsorption/desorption isotherm.^10^ In our opinion this hysteresis can be governed by the limiting uptake of condensate in the range of high *p*_1_/*p*_sat_, as well as by an contact angle hysteresis for an advancing/receding meniscus. Thus, we suggest that the equilibrium value of membrane permeance in the condensation regime should be the average between the values measured on the “adsorption” and “desorption” branches.

In the absence of capillary condensation – the region where *p*_1_/*p*_sat_<0.9 – the computed and measured permeances coincide nicely. Due to the pure gaseous flow through the entire membrane, heat transfer to the downstream boundary or the contact angle do not influence the membrane permeance. For the gaseous phase, the flow resistance is calculated as a combination of viscous and molecular flow. Hence, the flow resistance depends on the viscosity of the fluid, the pore diameter, and the correction factor *β* for molecular flow. The value of the correction factor *β* is not adjusted, and a fixed value of *β* = 9.05 based on theoretical considerations is used. The significant increase of the permeance with increasing upstream pressure is caused, at least according to the theoretical description, by condensation and the large pressure differences across curved interfaces between the liquid and the gaseous phases.

Under conditions where condensation occurs, the mass flow through a given membrane strongly increases with increasing upstream pressure. Fig. 6 shows computed pressure distributions where the fluid condenses and liquid flows through a part of the membrane. Blue color indicates liquid in Fig. 6. In the case depicted in Fig. 6(a) and (c), liquid flows between the upstream front of the membrane, *x* = 0, and a position of approximately 0.8 times the membrane thickness, *x*/*L* ≈ 0.8. From the pressure differences between the gaseous and the liquid phase at *x*/*L* = 0 and at *x*/*L* ≈ 0.8 one can infer that the curvature of the interface at *x*/*L* = 0 is smaller than the curvature of the interface within the membrane at *x*/*L* ≈ 0.8. The curvature of the interface within the membrane is determined by the contact angle, which is fixed. Hence, the pressure difference across the interface within the membrane always stays the same and is independent of the flow conditions. Conversely, the curvature of the interface at the upstream meniscus depends on the upstream condition of the fluid. The larger the upstream pressure, the smaller becomes the curvature of the interface and, hence, the smaller becomes the pressure difference at the upstream front of the membrane. Therefore, with increasing upstream pressure, the large pressure difference across the interface within the membrane must be balanced by an increasing pressure difference due to a larger mass flow rate through the membrane.

**Fig. 5:**
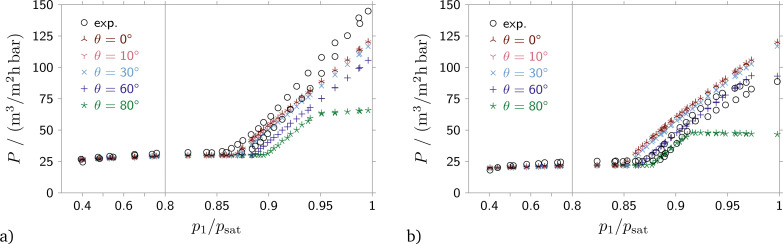
Permeance *versus* relative upstream pressure for a pore size of 40 nm, *ϕ* = 0.7. Data is shown for a different membrane than that used in Fig. 3. Flow of (a) isobutane and (b) freon 142b.

**Fig. 6:**
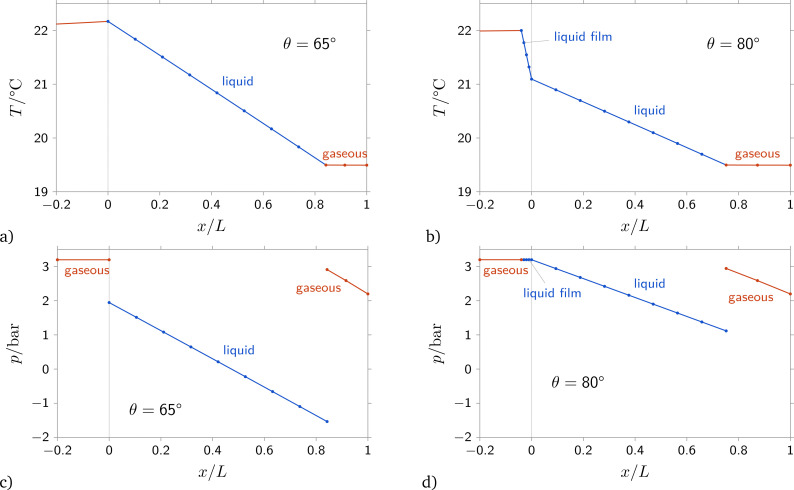
Distribution of pressure and temperature along the membrane for contact angles of *θ* = 65° and *θ* = 80°. Computed for isobutane, *T*_1_ = 22 °C, adiabatic b.c., *p*_1_ = *p*_sat_, *p*_1_ − *p*_2_ = 1 bar, pore size 40 nm.

The analysis of calculated values of gas permeance in capillary condensation regime using the suggested model and taking into account different values of wetting angle indicates a small decrease of membrane permeance and a slight increase of the minimum pressure at which capillary condensation occurs with an increase of the contact angle up to 60°, see Fig. 5 for a pore size of 40 nm and Fig. S3 and S5 in the ESI† for pore sizes of 18 and 60 nm. However, further increasing the contact angle to 80° results in changing the permeance *vs.* pressure dependence. A kink appears in the permeance *vs.* pressure dependence in the capillary condensation regime. This kink corresponds to the formation of a condensate film on the membrane surface, see Fig. 6(b) and (d). The liquid film formation at the membrane surface restricts the condensate permeation rate through the membrane, since there is now a fixed pressure difference between the plane interface of the liquid film and the meniscus within the membrane. Also, it should be noted that according to the agreement between experimental data and computed points, the contact angles for isobutane and 1-chloro-1,1-difluoroethane (freon 142b) differ. For butane, the contact angle is about 0°, while for freon 142b it is about 60°.

**Fig. 7:**
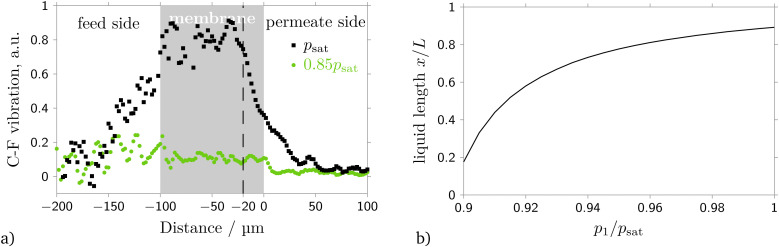
Fraction of membrane filled with liquid. (a) Dependence of signal intensity of the C–F vibration mode on the depth of focus of the excitation laser for upstream pressures of *p*_1_ = *p*_sat_ and *p*_1_ = 0.85*p*_sat_. (b) Computed position of liquid–gaseous interface for freon 142b, pore diameter 40 nm, (*T*_1_ − *T*_2_)/(*T*_1_ − *T*_2,adiabatic_) = 0.7 and upstream temperature 25 °C.

Confocal Raman microscopy has been utilized to prove the calculated distribution of condensate in the membrane. The signal intensity from the C–F vibration mode of freon 142b was measured depending on the depth of focus of the excitation laser (see Section 2, Fig. 1(b)). The obtained distributions of C–F vibration intensity at feed pressures of 0.85*p*_sat_ and *p*_sat_, in the absence and under capillary condensation conditions, respectively, are shown in Fig. 7(a). One can observe that under capillary condensation conditions the intensity of C–F vibration inside he membrane is then times higher than outside of the membrane. While in absence of capillary condensation the intensity of the signal inside and outside of the membrane is nearly the same. This can be explained due to the higher density of freon 142b inside the membrane, indicating the occurrence of capillary condensation. Therefore the signal distribution allows to determine the degree of pore filling with condensate. For a feed pressure equal to *p*_sat_ the signal intensity decreases rapidly at a depth of about 80 microns from the upstream side of the membrane. This value is in good agreement with the position of the liquid–gaseous interface calculated theoretically using the suggested model, see Fig. 7(b).

### 5.3 Properties of the suggested model

**Fig. 8:**
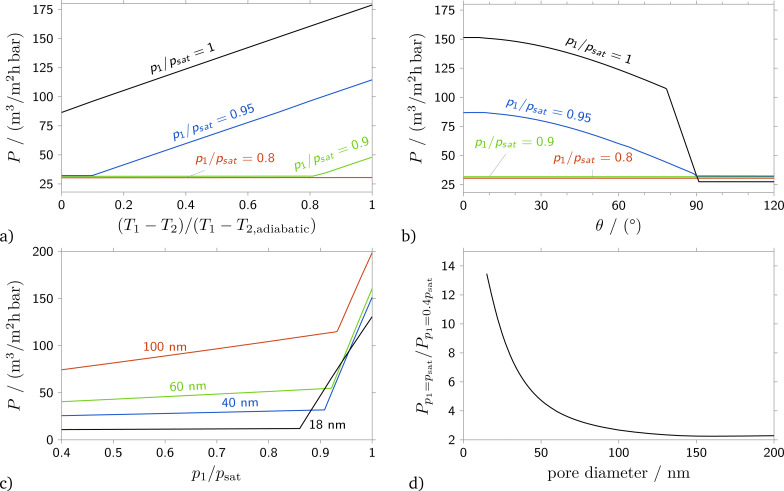
Dependence of permeance on (a) temperature difference for *θ* = 0°, and on (b) contact angle and (c) *p*_1_/*p*_sat_ for (*T*_1_ − *T*_2_)/(*T*_1_ − *T*_2,adiabatic_) = 0.7. (d) Permeance increase for *p*_1_ = *p*_sat_*versus p*_1_ = 0.4*p*_sat_. Computed for isobutane at *T*_1_ = 22 °C, *p*_1_ − *p*_2_ = 1 bar and a membrane with thickness 100 μm, porosity 0.15 and a pore diameter of 40 nm, if not stated otherwise.

Turning to a parametric study to evaluate the influence of contact angle and heat transfer coefficient on membrane permeance in the capillary condensation regime, Fig. 8 shows computations of the permeance for a number of *p*_1_/*p*_sat_ ratios depending on the temperature difference *T*_1_ − *T*_2_ at a fixed contact angle of 0°, Fig. 8(a), and depending on the contact angle for *T*_1_ − *T*_2_ = 0.7(*T*_1_ − *T*_2,adiabatic_), Fig. 8(b). A permeance of about 30 m^3^(STP) m^−2^ h^−1^ bar^−1^ corresponds to purely gaseous flow through the membrane with 40 nm pore diameter. For an upstream pressure of *p*_1_/*p*_sat_ = 0.8 and smaller, condensation does not occur, and there is always gaseous flow regardless of the downstream temperature, *cf.*Fig. 8(a). Also, the mass flow rate of gaseous flow does not depend on the temperature difference. For *p*_1_ = *p*_sat_, on the contrary, the permeance is for all temperature differences larger than the permeance according to gaseous flow, indicating capillary condensation. Under conditions where capillary condensation occurs, the permeance increases linearly with increasing temperature difference *T*_1_ − *T*_2_. The linear increase of the permeance is caused by the increase of the radius of curvature of the upstream meniscus. For instance, for *p*_1_/*p*_sat_ = 0.95, at (*T*_1_ − *T*_2_)/(*T*_1_ − *T*_2,adiabatic_)≈0.1, see Fig. 8(a), the fluid just condenses, and the temperature distribution is such that the radius of curvature of the upstream meniscus is nearly equal to the radius of curvature of the meniscus within the membrane. Hence, the pressure differences across these two menisci balance each other, *cf.*Fig. 2, and the mass flow rate is approximately equal to the mass flow rate for gaseous flow. With increasing temperature difference, the radius of curvature of the upstream meniscus increases, while the curvature of the meniscus within the membrane stays the same. The net effect is an increased pressure difference driving the flow. A temperature difference of (*T*_1_ − *T*_2_)/(*T*_1_ − *T*_2,adiabatic_) = 1 corresponds to the adiabatic downstream boundary condition, eqn (6), while *T*_1_ − *T*_2_ = 0 corresponds to the diabatic boundary condition, eqn (8). Changing the boundary conditions from adiabatic to diabatic reduces the permeance by more than 50%.

The influence of the contact angle *θ* on membrane permeance is shown in Fig. 8(b). With increasing contact angle, the permeance decreases, see the lines for *p*_1_/*p*_sat_ = 1 and *p*_1_/*p*_sat_ = 0.95. There is a kink in the dependence of permeance on contact angle, at *θ* ≈ 80° for *p*_1_ = *p*_sat_ and at a value close to 90° for *p*_1_/*p*_sat_ = 0.95. For contact angles smaller than the location of the kink, the vapor condenses at the upstream front of the membrane. For larger contact angles, a liquid film forms. Pressure and temperature distributions for a case without liquid film and a case with liquid film are shown in Fig. 6. For small contact angles, if the vapor condenses at the upstream front of the membrane, there is a curved meniscus at the upstream front of the membrane and a second meniscus within the membrane, where the condensate evaporates. The pressure difference over the condensate increases the mass flow rate through the membrane, and the pressure differences across the curved menisci partially balance each other. Conversely, if a liquid film forms, the upstream surface of the liquid is plane, and the pressure difference over the condensate is governed by the meniscus within the membrane, and hence the contact angle. Therefore, for small to moderate contact angles, the dependence of permeance on contact angle is not overly large. Differently, in a small range of contact angles close to 90°, visible in the line for *p*_1_/*p*_sat_ = 1, the permeance decreases linearly with increasing contact angle.

One last observation warranted by Fig. 8(b) concerns the permeance for *θ* > 90°. For *p*_1_/*p*_sat_ = 1 the fluid likely condenses, not due to capillary condensation, but caused by heat transfer due to the Joule–Thomson effect.^15,16^ A liquid film forms in front of the membrane, and the liquid evaporates at the upstream front of the membrane. There is a small, additional pressure difference at the upstream surface of the membrane that keeps the liquid film in place. Therefore, for *p*_1_ = *p*_sat_, the permeance is slightly smaller than for smaller upstream temperatures.

Fig. 8(c) and (d) present an analysis of the influence of the pore diameter on the membrane permeance. For membranes with the same porosity, obviously an increase in pore diameter results in an increase of gas permeance at pressures far from saturation. Also, the slope of permeance-pressure dependences increases with increasing pore diameter indicating the growth of viscous flow contribution. According to the Laplace equation, growth of the pore diameter leads to a shift of the minimum pressure for which condensation occurs.

The permeance pressure dependence in the capillary condensation regime becomes more interesting for various pore diameters. Due to the action of capillary pressure, which is larger for smaller pores, there might be a region of upstream pressures where the mass flow rate through a membrane with a smaller pore size is larger than the mass flow rate through a membrane with larger pores. In Fig. 8(c), in the range 0.9 < *p*_1_/*p*_sat_ < 0.93 the permeance for a membrane with pore diameter 18 nm is larger than the permeance for membranes with 40 and 60 nm pore diameter.

However, if we consider the dependence of the membrane permeance in the capillary condensation regime normalized to its Knudsen permeance (at feed pressure 0.4*p*_sat_), we can find that this value decreases significantly with increasing pore diameter from 15 for a pore diameter of 18 nm to 2.2 for membranes with pore diameters larger than 100 nm, see Fig. 8(d).

## 6 Conclusions

In the current work, we propose a model for description of the flow of vapors through nanoporous media with determined thickness, pore diameter, and porosity in the capillary condensation regime. This model takes into consideration heat transfer from the condensing to evaporating meniscus, different boundary conditions for the heat transfer from the environment to the membrane, and different wettability of the pore wall with condensate.

The influence of the main parameters of the suggested model on the calculated value of the membrane permeance was analyzed. The heat transfer to the membrane has a significant influence on its permeance – the variation of the heat transfer coefficient from zero (adiabatic boundary condition) to infinity (diabatic boundary condition) leads to less than half the vapor permeance through the membrane for a feed stream pressure equal to the saturation pressure. At the same time, a variation of the contact angle value in the range of 0–60° has a moderate influence – the permeance decreases by less than 15%. However, contact angles larger than 75–80° significantly suppress the membrane permeance in the capillary condensation regime.

Nevertheless, the agreement between experimental data and calculations using the suggested model with two extremum boundary conditions for the heat transfer coefficient – adiabatic and diabatic – is not satisfactory. Therefore, an appropriate boundary condition was found by measuring the permeate side temperature of the membrane. The permeate side temperature always lied at a fixed, constant fraction between the temperature of the environment and the temperature that would be expected from the ideal, adiabatic boundary condition. This boundary condition is equivalent to the heat flux from the environment to the membrane being proportional to the mass flux through the membrane. The obtained boundary condition allows an excellent fit of experimental data and computed results for condensate flow through anodic alumina membranes with different pore diameters for two different vapors, isobutane and freon 142b. Also, comparison of experimental and calculated data might indicate a different wettability of the membrane pore wall with isobutane and freon 142b.

Additional confirmation of the proposed model was obtained using confocal Raman spectroscopy, which allows to determine the amount of liquid present within the membrane. According to the theoretical description, for a feed pressure equal to the saturation pressure approximately 80% of the pore volume is filled with liquid, a value which is confirmed by Raman spectroscopy.

We believe that considering heat transfer processes and the temperature variation can aid to better understand capillary condensation and evaporation processes in the porous media, to predict membrane permeance in the capillary condensation regime, and to suggest the process conditions for attaining the highest membrane permeance-to-selectivity ratio.

## Author contributions

SP and DP: investigation, JS and TL: formal analysis, TL: software, DP and AE: validation, TL and DP: writing – original draft, TL, DP and AE: writing – review & editing.

## Conflicts of interest

There are no conflicts to declare.

## Supplementary Material

CP-025-D2CP04577J-s001
