# Supplementary material for: Influence of heat transfer and wetting angle on condensable fluid flow through nanoporous anodic alumina membranes
Source: Phys Chem Chem Phys. 2023 Jan 10;25(4):3240–50. doi: 10.1039/d2cp04577j (PMC9890497; doi:10.1039/d2cp04577j)
Supplement: CP-025-D2CP04577J-s001 [file CP-025-D2CP04577J-s001.pdf]

Electronic Supplementary Material (ESI) for Physical Chemistry Chemical Physics.
This journal is © the Owner Societies 2022

Supporting Information: Influence of heat transfer and wetting angle on condensable fluid flow through nanoporous anodic alumina membranes

Thomas Loimer,[*,†] Stepan K. Podgolin,[‡] Javad Sodagar-Abardeh,[†] Dmitrii Petukhov,[*,¶] and Andrei A. Eliseev[‡]

†*Institute of Fluid Mechanics and Heat Transfer, TU Wien, Austria*

‡*Department of Materials Science, Lomonosov Moscow State University, Russia*

¶*Department of Chemistry, Lomonosov Moscow State University, Russia*

E-mail: thomas.loimer@tuwien.ac.at; di.petukhov@gmail.com

Phone: +43 1 58801 32233

List of Figures

S1 Experimental cell. S-3

S2 Permeance versus relative upstream pressure for a pore size of 18 nm. Contact angle of 0° and boundary conditions: adiabatic, diabatic, $T_1 - T_2 = 0.7(T_1 - T_{2,\text{adiabatic}})$. Flow of (a) isobutane and (b) freon 142b. S-4

S3 Permeance versus relative upstream pressure for a pore size of 18 nm. Contact angles of 0°, 10°, 30°, 60°, and 80°, boundary condition $T_1 - T_2 = 0.7(T_1 - T_{2,\text{adiabatic}})$. Flow of (a) isobutane and (b) freon 142b. S-4

S4 Permeance versus relative upstream pressure for a pore size of 60 nm. Contact angle of 0° and boundary conditions: adiabatic, diabatic, $T_1 - T_2 = 0.7(T_1 - T_{2,\text{adiabatic}})$. Flow of (a) isobutane and (b) freon 142b. S-5

S5 Permeance versus relative upstream pressure for a pore size of 60 nm. Contact angles of 0°, 10°, 30°, 60°, and 80°, boundary condition $T_1 - T_2 = 0.7(T_1 - T_{2,\text{adiabatic}})$. Flow of (a) isobutane and (b) freon 142b. S-5

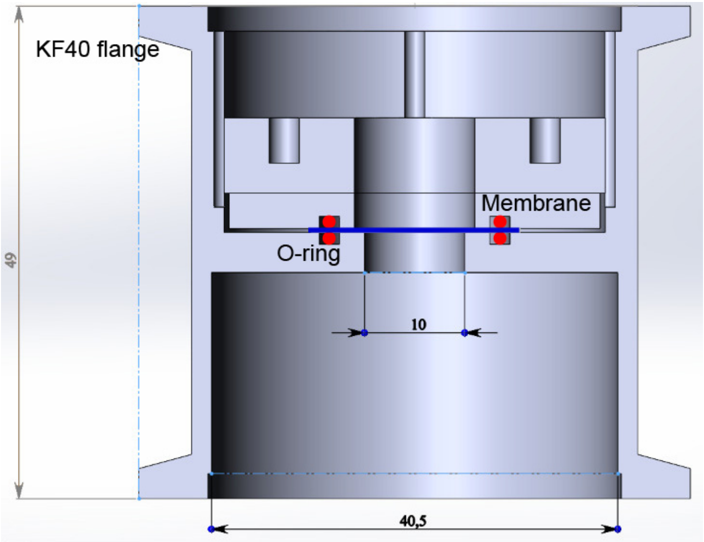

Figure S1: Experimental cell.

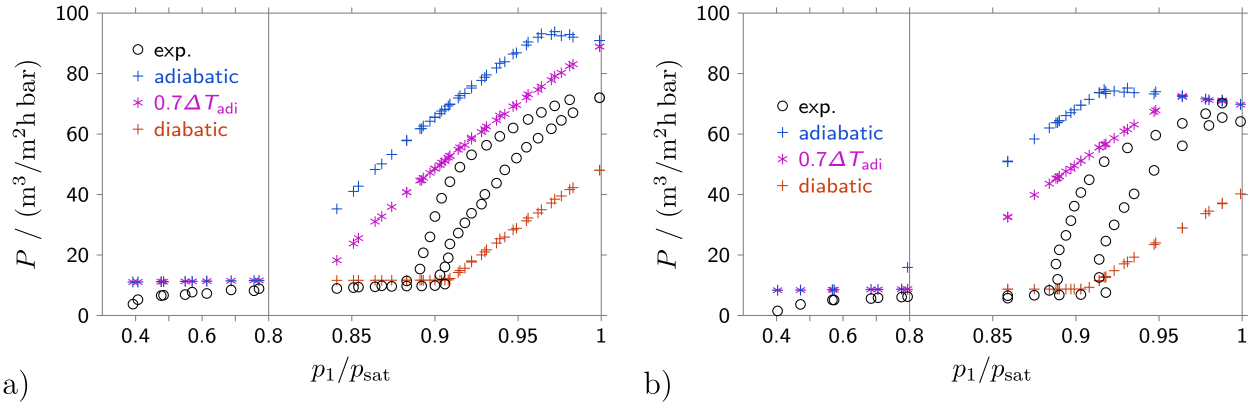

Figure S2: Permeance versus relative upstream pressure for a pore size of 18 nm. Contact angle of 0° and boundary conditions: adiabatic, diabatic, $T_1 - T_2 = 0.7(T_1 - T_{2,\mathrm{adiabatic}})$. Flow of (a) isobutane and (b) freon 142b.

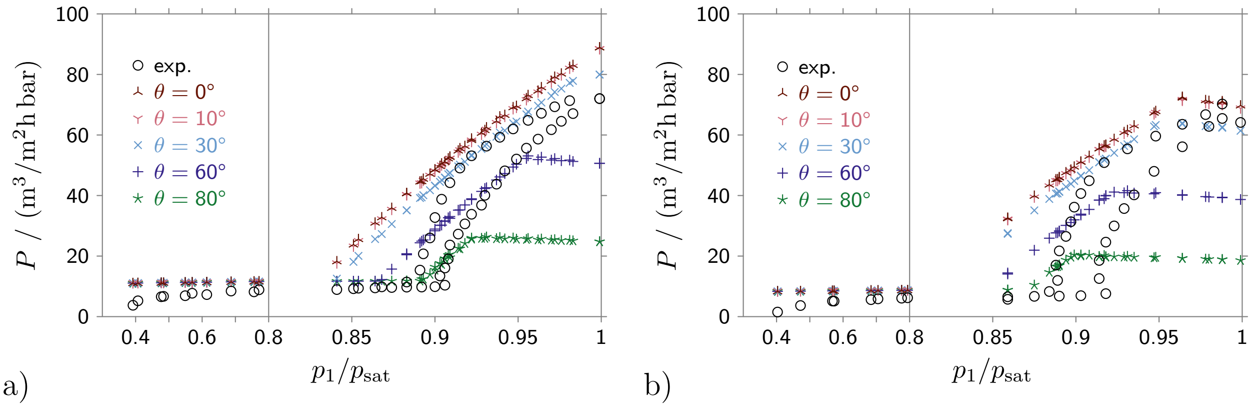

Figure S3: Permeance versus relative upstream pressure for a pore size of 18 nm. Contact angles of 0°, 10°, 30°, 60°, and 80°, boundary condition $T_1 - T_2 = 0.7(T_1 - T_{2,\mathrm{adiabatic}})$. Flow of (a) isobutane and (b) freon 142b.

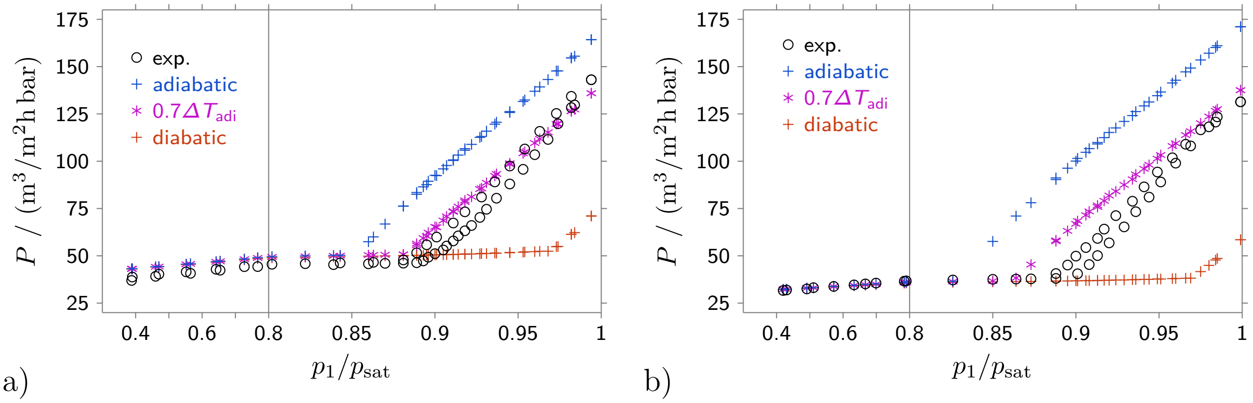

a) b)

Figure S4: Permeance versus relative upstream pressure for a pore size of 60 nm. Contact angle of 0° and boundary conditions: adiabatic, diabatic, $T_1 - T_2 = 0.7(T_1 - T_{2,\text{adiabatic}})$. Flow of (a) isobutane and (b) freon 142b.

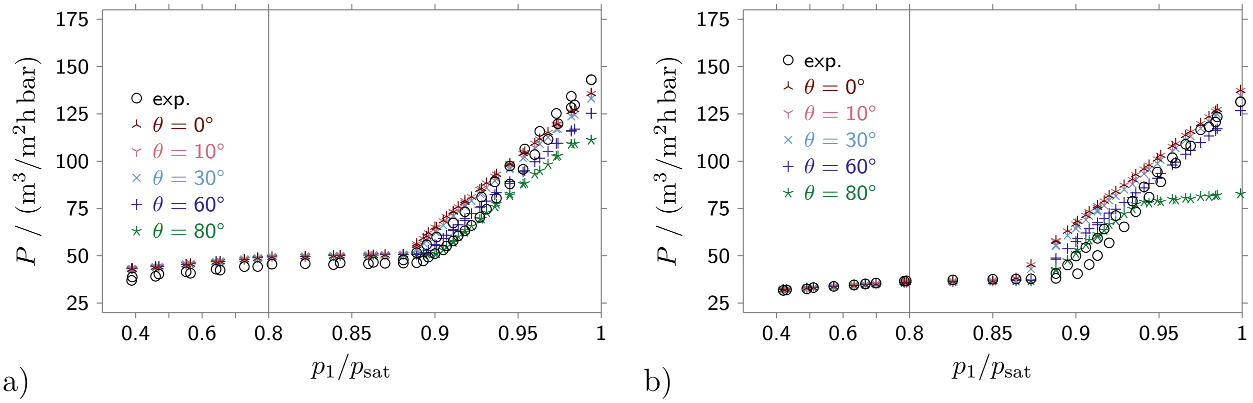

a) b)

Figure S5: Permeance versus relative upstream pressure for a pore size of 60 nm. Contact angles of 0°, 10°, 30°, 60°, and 80°, boundary condition $T_1 - T_2 = 0.7(T_1 - T_{2,\text{adiabatic}})$. Flow of (a) isobutane and (b) freon 142b.
